# Mutation of the elongin C binding domain of human respiratory syncytial virus non-structural protein 1 (NS1) results in degradation of NS1 and attenuation of the virus

**DOI:** 10.1186/1743-422X-8-252

**Published:** 2011-05-22

**Authors:** Claire P Straub, Wei-Har Lau, Faith M Preston, Madeleine J Headlam, Jeffery J Gorman, Peter L Collins, Kirsten M Spann

**Affiliations:** 1 The University of Queensland, Clinical Medical Virology Centre, QLD 4072, Australia; 2 Children's Hospital District, Sir Albert Sakzewski Virus Research Centre, QLD 4029, Australia; 3 Protein Discovery Centre, Queensland Institute of Medical Research, PO Royal Brisbane Hospital, Herston, Queensland, 4029, Australia; 4 Respiratory Virus Section, Laboratory of Infectious Diseases, National Institute of Allergy and Infectious Disease, National Institutes of Health, Bethesda, MD 20892

**Keywords:** RSV, NS1, attenuation

## Abstract

**Background:**

Human respiratory syncytial virus (RSV) is an important cause of lower respiratory tract disease in the paediatic population, immunocompromised individuals and the elderly worldwide. However, despite global efforts over the past several decades there are no commercially available vaccines. RSV encodes 2 non-structural proteins, NS1 and NS2, that are type I interferon antagonists. RSV restricts type I interferon signaling and the expression of antiviral genes by degrading STAT2. It has been proposed that NS1 binds to elongin C to form a ubiquitin ligase (E3) complex that targets STAT2 for ubiquitination and proteosomal degradation.

**Results:**

Here, we have engineered a live recombinant RSV in which the 3 consensus amino acids of the NS1 elongin C binding domain have been replaced with alanine (NS1F-ELCmut). Mutation of this region of NS1 resulted in attenuation of RSV replication in A549 cells to levels similar to that observed when the NS1 gene is completely deleted (ΔNS1). This mutation also resulted in moderate attenuation in Vero cells. Attenuation was correlated to intracellular degradation of the mutated NS1 protein. Time course analysis showed that mutant NS1 protein accumulated in cytoplasmic bodies that contained the lysosomal marker LAMP1. However lack of cleavage of LC3 suggested that autophagy was not involved. Induction of IFN-β mRNA expression also was observed in association with the degradation of NS1 protein and attenuation of viral growth.

**Conclusions:**

These results indicate that the elongin C binding region of NS1 is crucial for survival of the protein and that disruption of this region results in the degradation of NS1 and restriction of RSV replication.

## Background

Human respiratory syncytial virus (RSV) is the most common cause of pediatric viral bronchiolitis and pneumonia in infants and young children worldwide, and also causes severe respiratory infection in immunocompromised adults and the elderly [1,2]. Despite its world-wide importance, and several decades of research, there is still no vaccine or specific antiviral therapy for RSV disease [3]. RSV has a single-stranded negative-sense RNA genome, and belongs to the genus *Pneumovirus *of the family Paramyxoviridae [1]. The RSV genome encodes 11 proteins, including attachment and fusion proteins G and F, nucleocapsid-associated proteins N, P and L, transcription and RNA replication factors M2-1 and M2-2, the matrix M protein, small hydrophobic SH protein, and two non-structural proteins NS1 and NS2. The NS1 and NS2 proteins are dispensable for viral replication *in vitro*. However, ablation of either NS protein, or both, significantly attenuates the growth of RSV *in vitro *and *in vivo *[4-7].

Most viruses encode proteins that inhibit the innate immune response to viral infection and promote virus replication [8,9]. NS1 and NS2 of both bovine and human RSV are type I Interferon (IFN α/β) antagonists and target type I IFN induction and signaling [7,10-13]. Deletion of NS1, more so than NS2, from human recombinant (r) RSV (rRSVΔNS1) attenuates replication and results in an increase in the expression of type I IFN-α/β and type III IFN-λ, compared to wild-type (wt) rRSV [7]. However, deletion of both NS proteins (rRSVΔNS1/2) results in a greater induction of type I and type III IFN expression and attenuates rRSV to a greater extent than deletion of either single NS protein. Deletion of NS1 and/or NS2 also attenuates rRSV in Vero cells, which do not express type I IFN [6,7]. This suggests that NS1 and NS2 have additional functions, independent of the type I IFN response, that affect RSV replication. One such function is the suppression of early apoptosis (<18 h) in RSV-infected cells [14]. RSV induces both pro- and anti-apoptotic factors in A549 and primary epithelial cells [15]. The NS proteins, both individually and together, delay apoptosis and promote viral replication via an IFN-independent pathway [14]. RSV NS1 and NS1/2 deletion mutants enhance maturation of infected human dendritic cells, also suggesting that NS1, and to a lesser extent NS2, suppress DC maturation leading to a weakened immune response to infection [16].

The mechanisms by which NS1 and NS2 suppress the antiviral response are proving to be complex. RSV is known to degrade STAT2, which is required for the transcription of genes encoding a range of antiviral cellular factors [17-20]. Recently, a mechanism by which NS1 targets STAT2 for ubiquitination and proteasome-mediated degradation has been proposed. Elliot *et al.*, (2007), have identified consensus elongin C and cullin 2 binding sequences within NS1. They have described the potential of NS1 to bind directly to elongin C and act as an E3 ligase to target STAT2 to the proteasome for degradation.

NS1/2 deletion mutants are being developed as live-attenuated vaccine candidates. Preclinical studies in chimpanzees demonstrated that both ΔNS1 and ΔNS2 deletion viruses were substantially attenuated in the upper and lower respiratory tracts and induced significant resistance to challenge with wild-type virus [5,21,22]. Combination of the NS2 deletion with cold-passaged (cp) and temperature-sensitive (ts) mutations, resulting in the vaccine candidates rA2*cpts*248/404ΔNS2 and rA2*cpts*530/1009ΔNS2, proved to be overattenuating in seronegative children [3]. Evaluation of a ΔNS1 vaccine candidate is planned. However, virus that lacks NS1 replicates less efficiently *in vitro*. It would be advantageous to identify residues in NS1 that are involved in antagonising the IFN response, and if possible, to ablate these activities. To this end we sought to develop and characterize a live rRSV containing modification of one of the putative functional regions of NS1, namely the proposed elongin C binding domain [19].

This domain (VxxLxxxCxxxK) in NS1 does not conform exactly with the consensus sequence (VxxLxxxCxxx(A/I/L/V) of other elongin C-interacting proteins such as VHL and SOCS 1-3 [23,24]. For this reason we chose to modify only the 3 consensus residues (V, L and C) by substitution with alanine (A). Here we demonstrate that this region of NS1 is critical for the survival of the NS1 protein, and that rapid degradation of NS1 as a result of this mutation correlates with viral attenuation in both type I IFN-competent (A549) and -incompetent (Vero) cells.

## Materials and methods

### Cells and virus stocks

Vero (African green monkey), HEp-2a (human epithelial) and A549 (human type II alveolar epithelial) cells were grown in Opti-MEM (Invitrogen) containing 5% FBS (Sigma-Aldrich). Cells were incubated at 37°C in 5% CO_2_. Viral stocks were generated in Vero cells infected at a MOI of 0.1 PFU/cell and harvested 7 days later. The titre of viral stocks was determined by plaque assay. Infected cells were incubated at 37°C for 7 days under 0.8% methyl cellulose (Sigma) in OptiMEM with 2% FBS, and plaques visualized by immunostaining using a goat anti-RSV polyclonal antibody (Virostat) followed by horse-radish peroxidase-coupled anti-goat IgG antibodies and DAB peroxidase substrate (Sigma) as previously described [25].

### Construction of NS1F, NS1F-ELCmut, ΔNS1 cDNA clones

Construction of a cDNA copy of the RSV genome under the control of a T7 promotor has been described previously [26]. The full-length RSV cDNA used in the present study was D46/6120, which is based on strain A2 and contains a stabilizing 112 nt deletion of the downstream non-coding region of SH as well as several silent mutations in the last codons of the SH ORF [27,28]. The full-length cDNA clone was not mutated directly, rather a pGEM 7Z(+) plasmid containing the *Aat*II-*Xba*I fragment (leader, NS1, NS2 and part of the N genes) was generated and used for site directed mutagenesis (Stratagene Quikchange kit). A FLAG (Sigma) tag and diagnostic *Kpn*I site were introduced to the N-terminus of the NS1 ORF directly following the start codon using the oligonucleotide: 5'- GG TTA GAG ATG *GAC TAC AAG GAC GAC GAC GAC AAG *GGT ACC GGC AGC AAT TC-3' (FLAG tag in italics, *Kpn*I site underlined). The putative elongin C binding domain of NS1, also was mutated by site directed mutagenesis. The 3 consensus amino acids of the NS1 elongin C binding motif VxxLxxxC were mutated to alanine using the oligonucleotide: 5'-G TTT GAC AAT GAT GAA GCA GCA TTG GCA AAA ATA ACA GCC TAT ACT G-3' (alanine underlined). *Aat*II-*Xba*I mutated fragments, containing either the introduced FLAG tag within NS1, or the FLAG tag and the alanine substitution mutations, were introduced into the full-length antigenome cDNA D46/6120 using standard restriction enzyme digestion and T4 ligase (New England Biolabs) according to the manufacturer's instructions. The mutated cDNA clones were designated NS1F (FLAG tag) and NS1F-ELCmut (FLAG tag and alanine substitutions within the elongin C binding domain), and sequenced to ensure the presence of the mutations. A ΔNS1 cDNA clone, containing deletion of the NS1 open reading frame from D46/6120, was also engineered, by excising the entire NS1 gene, including the gene start and gene end signals, following the introduction of flanking *Pst*I sites (Stratagene).

### Recombinant RSV (rRSV) recovery

Mutated rRSVs were recovered using a modified transfection protocol described previously [26,29]. Briefly, 75% subconfluent BSR T7/5 cells were transfected in 6-well dishes simultaneously with 5 μg of antigenome plasmid (NS1F, NS1F-ELCmut or ΔNS1), 2 μg each of the support plasmids pTM1-N and pTM1-P, and 1 ug each of pTM1-L and pTM1-M2-1. Transfections were performed with Lipofectamine 2000 (Invitrogen) in OptiMEM without serum at 37°C. After 1 day the transfection medium was replaced with Glasgow's minimal essential medium (supplemented with 5% FBS and 1% L-glutamine) and the cells were expanded. After a further 4 days of incubation at 37°C, cell-medium mixtures were passaged onto fresh HEp-2a cells. Once cytopathic effects indicative of RSV infection were observed 5-7 days later, cell-medium mixtures were harvested and used to infect HEp-2a monolayers for three rounds of plaque purification. A stock of each recovered virus was then generated in Vero cells. The presence of the expected mutations and the absence of other spurious mutations were confirmed by RT-PCR and nucleotide sequencing of viral genomic RNA for all rRSVs generated.

### Growth of rRSV in vitro

Quadruplicate cell culture monolayers of A549 and Vero cells were infected with either wt RSV (D46/6120), NS1F, NS1F-ELCmut, or ΔNS1 at a MOI of 0.01 PFU/cell. Cell culture supernatants were collected daily for 5 days and the virus titres determined by plaque assay. Statistically significant differences in growth between rRSVs was detected by ANOVA

### Western Blot analysis

Triplicate cell monolayers were infected with either wt RSV, NS1F or NS1F-ELCmut at a MOI of 1 PFU/cell, or mock infected with media, and incubated at 37°C. At 24 h and 48 h p.i., cells were washed in PBS and lysed with RIPA buffer (25 mM Tris-HCl pH 7.6, 150 mM NaCl, 1% NP-40, 1% sodium deoxycholate, 0.1% SDS) containing protease inhibitors (Pierce). 20 μg total protein (BCA assay; Pierce) from each lysate was separated through pre-cast 12% polyacrylamide Bis-Tris gels (Invitrogen). Proteins were then transferred to nitrocellulose membranes and blocked in either 5% skim milk (Diploma)/PBS or 1% BSA (Sigma)/PBS containing 0.1% Tween 20. The blots were analysed using a goat anti-RSV antiserum (Virostat), or a rabbit antiserum raised against the C-terminal 13 amino acids of NS2, which reacts with both NS1 and NS2, and FLAG was detected using a mouse anti-FLAG IgG (Sigma). As a marker for autophagy, LC3 was detected using an antibody against LC3B (Cell Signaling Technology). β-actin was used as a loading standard and detected using a mouse anti-β-actin IgG (Sigma). Bound antibodies were visualized with species-specific IgG conjugated to either IR800 (Rockland Inc), or HyLyte680 (AnaSpec) and a Li-Cor Odyssey scanner. RSV protein expression was quantified using Odyssey™ densitometry software and statistical differences identified using Students t-test.

### RNA extraction and PCR

Cells were infected at a MOI of 1 with either NS1F, NS1F-ELCmut, ΔNS1, or mock infected with media, and harvested 24 h p.i., Total RNA was isolated using TRIzol (Invitrogen) and the RNeasy total RNA isolation kit (QIAGEN). RT-PCR was performed using RSV-specific primers and the PCR products sequenced also using RSV-specific primers. For quantitiative (q) PCR, RT was performed with oligo (dT)_20 _primers (Invitrogen) to select mRNA from the total RNA preparations. qPCR was performed using dual-labeled probe and primer sets as previously described [7] to quantify β-actin and IFN-β. PCR primers and dual-labeled probes to quantify NS1 and N mRNA were designed using Primer 3 http://frodo.wi.mit.edu/primer3/ software. qPCR was performed using QuantiTect™ reagents (QIAGEN) and the Rotogene 3000 (Roche). The fold increase in target expression compared to mock-infected was calculated using the 2^-ΔΔCt ^formula.

### Immunofluorescence

Confluent monolayers of Vero cells were cultured on coverslips and infected with either NS1F, NS1F-ELCmut or ΔNS1 at a MOI of 1 PFU/cell, or mock infected with media. At specific times, cells were fixed with 4% formaldehyde (ProSciTech) for 30 min and permeabilized with 0.1% Triton X-100 for 10 min. Primary and secondary antibodies were diluted in 1% BSA/PBS. NS1 and other RSV proteins were detected using antiserum also used for western blot anlaysis (above). Cellular organelles were detected using monoclonal antibodies to EEA, GM130, LAMP1 (BD Transduction Labs), and PDI (Invitrogen). Secondary antibodies, anti-mouse and anti-rabbit specific IgG were conjugated to Alexa Fluor 488 or 594 (Invitrogen). Following antibody incubation and washes, coverslips were mounted on glass microscope slides using ProLong Gold mounting medium (Invitrogen) and visualized using a Nikon Eclipse E3600 fluorescence microscope. Images were collated using adobe Photoshop software. Statistical differences in protein detection were identified using Student's t test.

## Results

### Insertion of a FLAG tag and amino acid substitutions in the NS1 protein of recombinant (r)RSV

**Figure 1:**
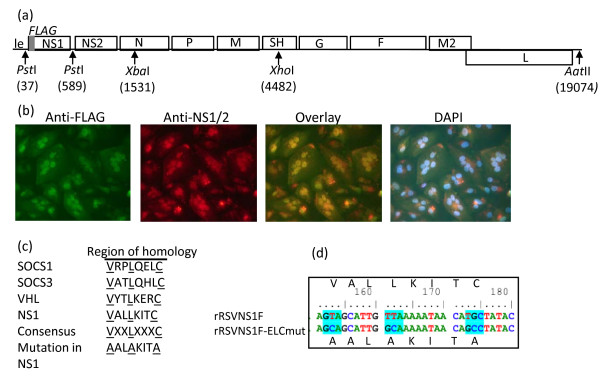
**Generation of rRSVs with a FLAG tag and mutations in NS1**. (a) The full-length rRSV plasmid D46/6120 showing restriction enzyme sites used to introduce mutations (see Materials and Methods) and the position of a FLAG tag (grey bar) introduced to the N-terminus of NS1 (NS1F virus). Le = leader region. (b) Recovered NS1F virus was analysed by immunofluorescence in Vero cells by dual labelling with anti-FLAG monoclonal antibody (green) and anti-NS1/2 polyclonal antiserum (red). Nuclei were stained with DAPI and overlay images generated using Photoshop. (c) The 3 consensus residues of the NS1 elongin C binding domain (underlined) were replaced with alanine and the resulting cDNA was used to generate the NS1F-ELCmut virus. (d) Nucleotide substitutions (blue) that changed the 3 consensus residues to alanine in the recovered NS1F-ELCmut virus.

For the present study, the wild-type (wt) rRSV construct D46/6120, based on the A2 strain, was modified to express a FLAG tag at the N-terminus of NS1 (NS1F; Figure 1a). This was to allow differentiation between NS1 and NS2, as the available NS-specific antiserum does not differentiate between NS1 and NS2 [30]. Immunofluorescence of NS1F-infected Vero cells demonstrated 100% co-localisation between FLAG and NS1/NS2 (Figure 1b). The NS1F clone was then modified to replace the consensus residues of the elongin C binding motif of NS1 with alanine residues (Figure 1c) to generate the NS1F-ELCmut virus, which was sequenced in its entirety and confirmed to contain the alanine replacements (Figure 1d). We also generated a ΔNS1 virus by first introducing *PstI *sites flanking NS1, and then using the *PstI *sites, to delete the entire NS1 gene (Figure 1a).

### Mutations in the elongin C binding motif reduced the replication of rRSV in vitro

**Figure 2:**
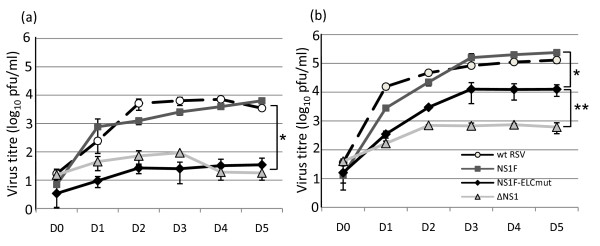
**The growth kinetics of NS1-mutated viruses**. Single step growth curves for the NS1F, NS1F-ELCmut and ΔNS1 viruses compared to wt RSV in (a) A549 and (b) Vero cells. The cell supernatants of quadruplicate cultures were collected daily and viral titres were determined by plaque assay. The mean titres (log_10 _pfu/ml) are shown with standard error. In A549 cells, NS1F-ELCmut and ΔNS1 were significiantly attenuated in comparison to both NS1F and wt RSV (*P < 0.01). In Vero cells, NS1F-ELCmut was attenuated in comparison to NS1F and wt RSV (* P < 0.01), and ΔNS1 was further attenuated in comparison with NS1F-ELCmut (** = P < 0.01).

Single step growth curve experiments, performed over 5 days post-infection (p.i.), in both Vero and A549 cells, demonstrated that the introduction of the FLAG tag to the N-terminus of NS1 (NS1F) did not affect viral growth *in vitro *compared to wt RSV (D46/6120; Figure 2a and 2b). By day 3 p.i. in both A549 and Vero cells, there was no significant difference in the titre of virus shed from NS1F and wt RSV-infected cells. Replacement of the 3 consensus residues within the elongin C binding motif of NS1 with alanine residues resulted in attenuation of the virus in comparison to both wt RSV and NS1F. In A549 cells, NS1F-ELCmut was significantly restricted in growth by 180-fold compared to NS1F at 5 days pi (P < 0.01). Deletion of the entire NS1 protein (ΔNS1) also significantly restricted growth as expected (P < 0.01). NS1F-ELCmut appeared to be more attenuated than ΔNS1 in A549 cells between days 0 and 3 pi, as the titre of shed virus was marginally significantly different (P < 0.05). However, given that the viral titre on D0 was higher for ΔNS1 than for NS1F-ELCmut, this is likely to not be biologically significant. (Figure 2a). The ΔNS1 growth curve in A549 cells is slightly atypical in that there is a reduction in shed virus on days 4 and 5 p.i. This was observed in several independent experiments (data not shown), and may be due to antiviral responses dampening shed virus. In Vero cells, NS1F-ELCmut replication was also significantly attenuated compared to NS1F and wt RSV (P < 0.01) over the 5 days, however, was less attenuated than in A549 cells, with a 20-fold reduction by 5 days pi. Deletion of the entire NS1 (ΔNS1) restricted growth by a further 20-fold in Vero cells (P < 0.01 compared to NS1F-ELCmut;Figure 2b).

### Mutation within the elongin C binding motif reduced the expression of NS1 protein

**Figure 3:**
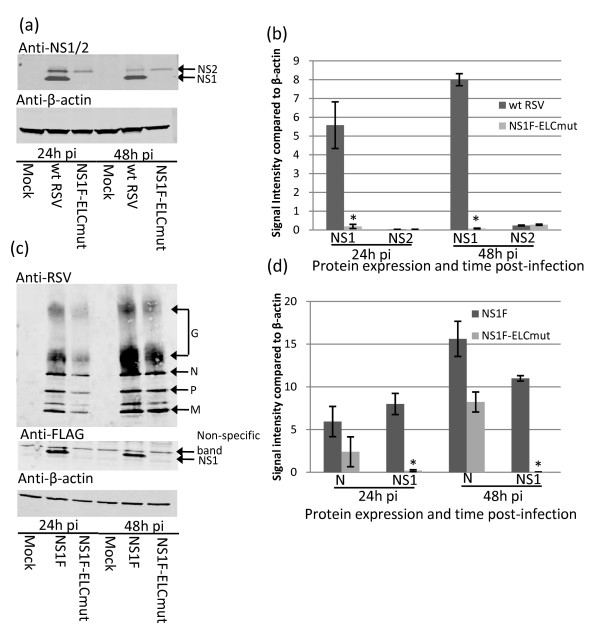
**The effect of mutation on NS1 and NS2 expression**. (a) Vero cells were infected with wt RSV, NS1F-ELCmut, or mock infected, and cells were lysed in RIPA buffer 24 h and 48 h p.i. Equivalent amounts of protein for each sample were separated by SDS-PAGE and analysed by Western blot using anti-NS1/2 polyclonal antiserum. Representative of three separate experiments. (b) Densitometric quantification of NS1 and NS2 expression in comparison to β-actin for triplicate wt RSV and NS1F-ELCmut-infected Vero cell cultures. NS1F-ELCmut infected cells expressed significantly less NS1 than wt RSV (* *P *< 0.001). NS2 expression was not different for the 2 viruses. (c) NS1F, NS1F-ELCmut and mock-infected cell lysates were probed with anti-FLAG to detect tagged NS1 protein and anti-RSV polyclonal antiserum to detect other RSV proteins. Representative of three separate experiments. (d) Densitometric quantification of NS1 and N expression in comparison to β-actin for triplicate NS1F and NS1F-ELCmut-infected Vero cell cultures. Cells infected with NS1F-ELCmut expressed significantly less NS1 than cells infected with NS1F (* *P *< 0.05).

We investigated the possibility that the 3 alanine substitutions within the elongin C binding motif of NS1 had reduced the expression of NS1 using Western Blot analysis. The expression of NS1 in Vero cells infected with NS1F-ELCmut was significantly reduced at 24 h and 48 h p.i. compared to cells infected with wt (D46/6120) RSV (*P *< 0.001; Figure 3a and 3b). NS2 expression was not affected by the mutations within the elongin C binding domain of NS1 (Figure 3a and 3b). The significant reduction of NS1 expression by NS1F-ELCmut also was observed by Western Blot when NS1 was detected using the FLAG tag (Figure 3c, middle panel). The non-specific band detected on this blot (Figure 3c, middle panel) is the same size as NS2, however appears in both mock-infected and viral-infected samples and is therefore not NS2. RSV infection in mock, NS1F and NS1F-ELCmut-infected cells was detected using anti-RSV polyclonal antibody (Figure 3c, upper panel). Quantification of N protein expression demonstrated that the NS1F-ELCmut virus was attenuated at 24 h and 48 h p.i. compared to NS1F virus, although the differences in expression were marginally significiant (*P *= 0.046; Figure 3d). In contrast, the expression of NS1 by the NS1F-ELCmut virus compared to the NS1F virus was significantly reduced (*P *= 0.005 at 24 h p.i., and 0.0034 at 48 h p.i.). This indicated that for the NS1F-ELCmut virus, NS1 protein expression was reduced more than would be expected due to restriction of viral replication alone.

### The mutations in the elongin C binding domain caused NS1 to accumulate in cytoplasmic bodies

**Figure 4:**
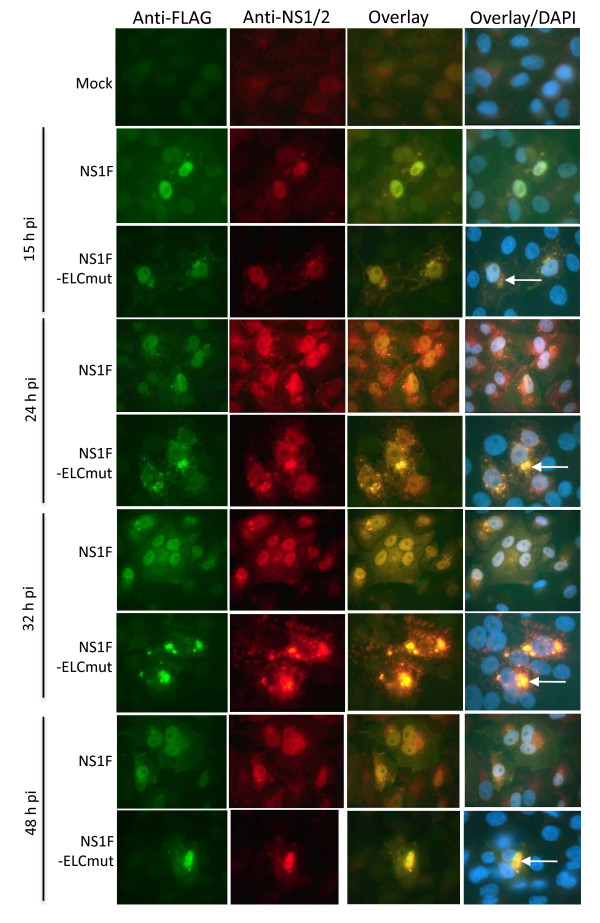
**The change in the intracellular distribution of mutated NS1 protein**. Vero cells were infected with NS1F, NS1F-ELCmut, or mock infected and were fixed at 15 (mock fixed at 15 h alone), 24, 32 and 48 h p.i. NS1 (white arrows) was detected by dual labelling using anti-FLAG (green) and anti-NS1/2 (red) antibodies. Nuclei were stained with DAPI and overlay images generated using Photoshop. Representative of 3 separate experiments.

Western Blot analysis of infected cells indicated that the NS1 expressed by NS1F-ELCmut was reduced, to undetectable levels. The expression of NS1 protein over time was investigated by immunofluorescence of Vero cells infected with NS1F or NS1F-ELCmut (Figure 4). NS1 was detected in only a small percentage of NS1F-ELCmut-infected cells (<3% at any time p.i.). In Vero cells infected with NS1F, NS1 was detected in both the cytoplasm and nucleus from 15 h to 48 h p.i. Tracking of NS1 expression over 48 h p.i. in cells infected with NS1F-ELCmut showed that NS1 was detected in the nucleus at 15 h and 24 h p.i., but not at 32 h or 48 h pi. From 15 h p.i., NS1 was observed to accumulate in the cytoplasm as distinct bodies (Figure 4, arrows). These data show that mutation within the elongin C binding motif of NS1 dramatically alters its cellular localization over time and induces the accumulation of NS1 within cytoplasmic bodies. A549 cells also were infected with NS1F and NS1F-ELCmut and stained for immunofluorescence detection of NS1 over 48 h pi. However, NS1 was not detected at any time in the NS1F-ELCmut-infected A549 cells.

### NS1F-ELCmut induced an increase in IFN-β induction

**Figure 5:**
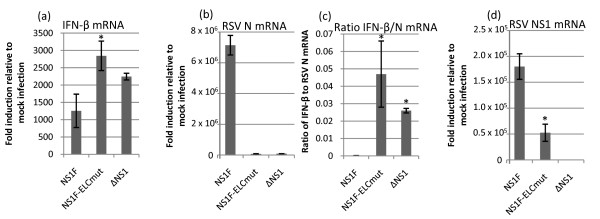
**NS1 mutation affects the expression of IFN-β, and RSV N and NS1 mRNAs**. A549 cells were infected with either NS1F, NS1F-ELCmut, ΔNSI, or mock infected, and total RNA was extracted 24h p.i. The levels of mRNA encoding (a) IFN-β or (b) RSV N were measured by RT-qPCR. (c) The ratio of IFN-β to RSV N mRNA was calculated. (d) The expression of NS1 mRNA was also measured by RT-qPCR. The level of expression of each mRNA was calculated using the 2 ^-ΔΔCt ^formula in relation to mock-infected samples, and was normalised to β-actin. The ratio of IFN-β/N mRNA was significantly higher in cells infected with NS1F-ELCmut and ΔNS1F compared to NS1F (* P < 0.01, ** P = 0.038).

We investigated the potential correlation of viral attenuation in A549 cells with an increase in the production of type I interferon (IFN). We quantified the levels of IFN-β, RSV N and RSV NS1 mRNA expression in relation to β-actin in A549 cells infected with NS1F, NS1F-ELCmut, or ΔNS1 at a MOI of 1 PFU/cell, or mock infected and harvested 24 h p.i. (Figure 5). We chose to investigate IFN-β mRNA expression and not IFN-α mRNA expression, as previous studies have shown than deletion of NS1 (ΔNS1) from rRSV has a greater effect on IFN-β expression than IFN-α expression [7]. Expression of mRNA was quantified as a fold increase compared to mock-infected cells. We found that IFN-β mRNA was elevated in cells infected with either NS1F-ELCmut or ΔNS1 compared to cells infected with NS1F (Figure 5a). However, this increase was only marginally statistically significant for NS1F-ELCmut (*P *= 0.038). In contrast, the levels of N mRNA in cultures infected with NS1F-ELCmut and ΔNS1F were greatly reduced compared to NS1F, reflecting their attenuation. Given the severe attenuation of NS1F-ELCmut and ΔNS1 in A549 cells, the amount of FN-β induction was interpreted relative to the level of viral replication, as represented by N mRNA, in these cells. When the ratios of IFN-β/N mRNA were compared, the rations for NS1F-ELCmut and ΔNS1 were significantly (*P *< 0.01) greater than for NS1F. The difference in IFN-β/N ratio between NS1F-ELCmut and ΔNS1 was not significant (Figure 5c). We also measured levels of NS1 mRNA (Figure 3d), and observed significantly lower levels in NS1F-ELCmut-infected A549 cells than in NS1F-infected A549 cells (*P *< 0.01), which correlated to differences in viral growth. We also confirmed that NS1 mRNA was not expressed by the ΔNS1 virus, as expected.

### Mutations in the elongin C binding motif of NS1 resulted in NS1 accumulation within lysosomes

**Figure 6:**
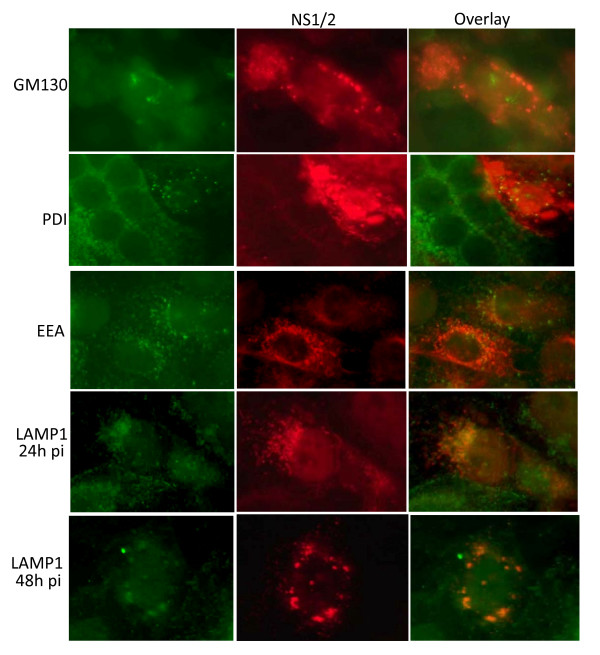
**The accumulation of mutated NS1 in cytoplasmic bodies**. Mutated NS1 protein accumulated in cytoplasmic bodies that did not co-localise with markers for *cis *-golgi (anti-GM130), ER (anti-PDI), or endosomes (anti-EEA-1), and did co-localise with a marker for lysosomes (anti-LAMP1). Vero cells infected with NS1F-ELCmut were fixed 24 h p.i. and dual labelled with markers for the indicated organelles (green) and anti-NS1/2 polyclonal antiserum (red). Overlay images were generated using Photoshop.

We investigated the identity of the cytoplasmic bodies containing the mutant NS1 using co-localisation of antibodies to NS1/2 and other cellular organelle markers. NS1 did not co-localise with the *cis*-golgi marker GM130 (golgi marker 130), the endoplasmic reticulum marker PDI (protein disulfide isomerase), or the endosome marker EEA (early endosome antigen 1). Co-localisation was observed with lysosome-association membrane protein 1 (LAMP1), a marker for lysosomes (Figure 6). At 24 h p.i., small NS1-positive cytoplasmic bodies were positive for LAMP1, indicating co-localisation of lysosomes and NS1. At 48 h p.i., larger NS1-positive cytoplasmic bodies were commonly observed, and LAMP1 detection associated with these larger, perinuclear bodies suggested that NS1 was localized within autolysosomes. These observations indicated that NS1 protein expressed by NS1F-ELCmut, was sequestered within cytoplasmic vesicles and degraded via a lysosomal pathway. There was no evidence of co-localisation of LAMP1 and NS1 protein expressed by NS1F (not shown).

**Figure 7:**
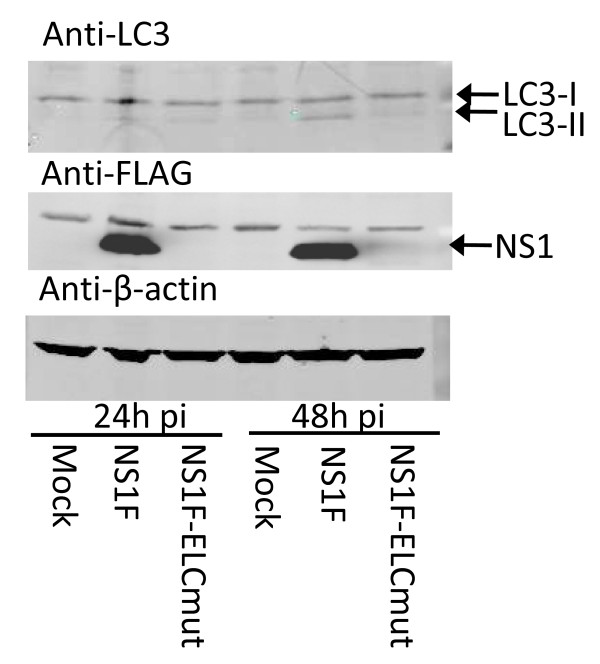
**The accumulation of mutated NS1 protein within lysosomes does not involve autophagy**. Vero cells were infected with either NS1F, NS1F-ELCmut, or mock infected with media, and cells were lysed in RIPA buffer 24 h and 48 h p.i. Equivalent amounts of total protein were separated by SDS-PAGE and analysed by Western blot. The active (LC3-II) and constitutive (LC3-I) forms of LC3 were detected using anti-LC3B polyclonal antibody. NS1 was detected using anti-FLAG monoclonal antibody. Representative of three independent experiments.

Autolysosomes are formed as part of the autophagy pathway. The constitutive cellular protein LC3, is processed proteolytically into LC3-II, which associates with the autophagosomal membrane and is widely used as a marker for autophagy. LC3-II was identified in NS1F-infected Vero cells, but not NS1F-ELCmut-infected Vero cells by 48 h p.i. (Figure 7). Therefore the association of NS1 with lysosomes does not appear to involve autophagy.

## Discussion

It is well documented that both of the non-structural proteins of RSV, NS1 and NS2, are type I IFN antagonists and target both the induction and signaling pathways. RSV mediates type I IFN signaling by degrading STAT2. Both NS1 and NS2 have been documented to play a role in this response [17-20]. Elliot *et al. *(2007) demonstrated that NS1 contains a consensus elongin C binding domain, and can form an elongin C-cullin-SOCS box-type E3 ubiquitin ligase. This may provide a mechanism by which RSV targets STAT2 for proteosomal degradation. The putative elongin C binding site within NS1, as described by Elliot *et al*, (2007), is VxxLxxxCxxxK, which differs in the fourth conserved residue from the consensus motif of VxxLxxxCxxxA/I/L/V [23,24]. Due to the variability of the final amino acid, we chose to replace the 3 residues with full homology, V, L and C, with alanines (A), as they are likely to be the most critical for function.

The resulting live rRSV NS1F-ELCmut was attenuated in both type I IFN competent (A549) and -incompetent (Vero) cells. This attenuation was similar to that observed when NS1 was ablated entirely (ΔNS1) in A549 cells, suggesting that mutation of the elongin C binding domain affected viral growth nearly as much as complete deletion of NS1. In fact, in A549 cells, mutation of the elongin C binding region affected NS1 expression such that it was never detected by western blot analysis or immunofluorescence. This suggests that the attenuation of NS1F-ELCmut and ΔNS1' were similarly due to the significant reduction or absence of NS1 protein expression respectively. Both NS1F-ELCmut and ΔNS1 also induced similarly increased levels of IFN-β mRNA, which correlated to viral attenuation and the absence or significant reduction of NS1 protein. The effect of deletion of NS1 on the replication of live rRSV is well documented [6,7,21,22], as is the correlating increase in the type I IFN response [7,17,18].

More insight into the effect of mutations within the elongin C binding domain of NS1 was gained using Vero cells. In these cells, the mutations in the elongin C binding motif of NS1 did lead to NS1 degradation, but the effect was delayed. Thus, NS1 was detected by immunofluorescence up to 48 h p.i. The presence of detectable NS1 early in infection and the slower rate of NS1 degradation in Vero cells, correlated to moderate attenuation of NS1F-ELCmut to levels between those of NS1F and ΔNS1. It has been observed previously that deletion of the NS proteins results in attenuation of RSV in type I IFN-incompetent Vero cells [6,7].

In Vero cells infected with NS1F-ELCmut, NS1 was degraded via a lysosomal-directed pathway. From 15 h pi to 48 h pi, there was a shift in the location of NS1 from being both nuclear and diffusely cytoplasmic, to being completely located within LAMP1-positive cytoplasmic bodies. The co-localisation of NS1 and LAMP1 suggested that NS1 was associated with lysosomes/autolysosomes and that degradation of NS1 may occur via autophagy. Autophagy involves the sequestration of cytoplasmic cellular material into double-membrane bound autophagosomes, which then fuse with lysosomes to form autolysosomes for degradation of intracellular materials [31]. Interactions between autophagy and viral infection have been documented for many viruses [31]. However, little is documented concerning autophagy during RSV infection. RSV-induced autophagy in dendritic cells has been shown to promote antiviral cytokine responses [32]. During autophagy LC3 is activated and recruited to the surface of autophagosomes. Activation of LC3, as demonstrated here in NS1F-infected cells, shows that RSV does induce autophagy in epithelial cells. The accumulation of LAMP1-positive bodies in the NS1F-ELCmut-infected cells suggested that autophagy may have been induced. However, the lack of LC3 activation by NS1F-ELCmut by 48 h p.i. indicates that the association of NS1 with lysosomes is not a consequence of increased autophagy. The lack of activated LC3 in NS1F-ELCmut-infected cells compared to NS1F-infected cells is most likely correlated with the reduced replication of the NS1F-ELCmut virus.

It is possible that the mutations introduced into the elongin C binding motif of NS1 caused misfolding of the protein leading to degradation. The tertiary structure is not known and therefore we were not able to model these mutations to assist with protein engineering. There is one report of a method for purifying rNS1-HIS^6 ^from *E. coli *and some information concerning secondary structure of NS1 [33]. However, functional regions have not yet been mapped and the consequences of changes in secondary structure on NS1 function are not known. Protein structure prediction using Protein Predict [34] indicated that this motif may be a beta strand (weak prediction), and that the alanine substitutions induce a predicted change to an alpha helix (results not shown). Given the increased accuracy of secondary predictions [35] it is reasonable to suspect that the introduced mutations indeed changed the local character of the secondary structure. However, whether this resulted in global misfolding leading to degradation is unknown.

Proteasomes and lysosomes represent the main proteolytic pathways in mammalian cells. Misfolded proteins are usually targeted for degradation via proteosomes [36], as the proteosomal pathway requires substrates to be unfolded to enter the narrow catalytic core [37]. Lysosomes, in contrast, fuse to vesicles containing proteins for degradation that are not necessarily misfolded. Thus, the finding that degradation of NS1 occurred via a lysosome-driven pathway rather than a proteosomal pathway in Vero cells suggests that the mutations in NS1 may not have resulted in global unfolding. Instead, it may be that the mutated NS1 protein became targeted for degradation as a consequence of its failure to associate with the E3 ligase complex. Although we attempted to investigate binding of NS1 to elongin C, by using the FLAG tag to immunoprecipitate NS1-associated proteins, the degradation of NS1 made this difficult. Hence we cannot directly confirm or deny binding of NS1 to elongin C.

The initial purpose of engineering mutations within the elongin C biding domain of NS1 was to investigate if NS1 did form an E3 ligase complex to degrade STAT2 in a live virus infection, and to alter this function, such that STAT2 degradation was reduced during RSV infection. Due to the rapid degradation of NS1 by these mutations we were not able to demonstrate the effect of these mutants on STAT2 within whole cell populations. We did perform infection experiments in A549 cells and identified isolated infected cells by immunofluorescence 24 h after infection. We found that STAT2 was degraded in NS1F-infected cells, as expected, and not degraded in NS1F-ELCmut- and ΔNS1-infected cells (additional file 1). This, however, was most likely the result of a reduction or lack of NS1 expression, not the result of mutations within NS1.

There has been considerable effort over the last 30 years to develop a vaccine for human RSV and currently live attenuated RSVs may offer the best candidates [3]. As deletion of NS1 or NS2 is attenuating and results in elevated type I IFN-mediated antiviral responses each deletion is being considered for inclusion in live-attenuated vaccine candidates. Modification or deletion of specific functional regions that inhibit the antiviral response may provide improved mutants in which the interferon antagonist activities have been ablated while providing improved growth. Alanine replacement of the conserved residues within the elongin C binding domain of NS1, however, resulted in attenuation similar to full deletion of NS1 in A549 cells. It is therefore unlikely that this present set of mutations will be useful in vaccine development. The data presented here do suggest, however, that this region is critical to the survival of NS1 and may be a potential target for antiviral agents. NS1 has proven to be a fruitful target for anti-RSV therapy. RNAi using nanoparticle delivery of siRNA targeting NS1 was used successfully to protect against infection and to clear an existing RSV infection in Fischer 344 rats [38]. A specific antiviral molecule that disabled elongin C binding and degraded NS1 may prove effective for clearing RSV infection.

## Conclusions

Mutations within the elongin C binding region of NS1 caused rapid degradation of NS1 protein, most likely via sequestration in lysosomes. This degradation of NS1 caused a correlated attenuation of RSV replication and an increase in the expression of IFN-β mRNA. Although this mutation is over-attentuated for inclusion in a live rRSV vaccine candidate, this region of NS1 is crucial for the survival of NS1 protein and may be a target for future antiviral compounds.

## Authors' contributions

KMS, JJG and PLC conceived of the study. KMS, CPS and WHL made the rRSVs and performed most experiments. FMP performed to qPCR experiments. MJH and JJG advised the mutations and MJH performed the protein prediction. KMS and CPS analysed the data and wrote the manuscript with assistance from JJG, MJH and PLC. All authors read and approved the final manuscript

## Competing interests

The authors declare that they have no competing interests.

## Supplementary Material

Additional file 1 **Immunofluorescence detection of STAT2 in infected A549 cells**. A549 cells were infected with NS1F, NS1F-ELCmut, ΔNS1 or mock infected and fixed 24 h post-infection. STAT2 (green) and RSV (red) were detected using specific antibodies. Nuclei were stained with DAPI and overlay images generated using Photoshop. STAT2 was degraded in NS1F-infected cells. STAT2 was not degraded in NS1F-ELCmut or ΔNS1-infected cells. Arrows = infected cells. Click here for file
