# Supplementary figures and images for: Mutation of the elongin C binding domain of human respiratory syncytial virus non-structural protein 1 (NS1) results in degradation of NS1 and attenuation of the virus
Source: Virol J. 2011 May 22;8:252. doi: 10.1186/1743-422X-8-252 (PMC3121706; doi:10.1186/1743-422X-8-252)

## Slide 1
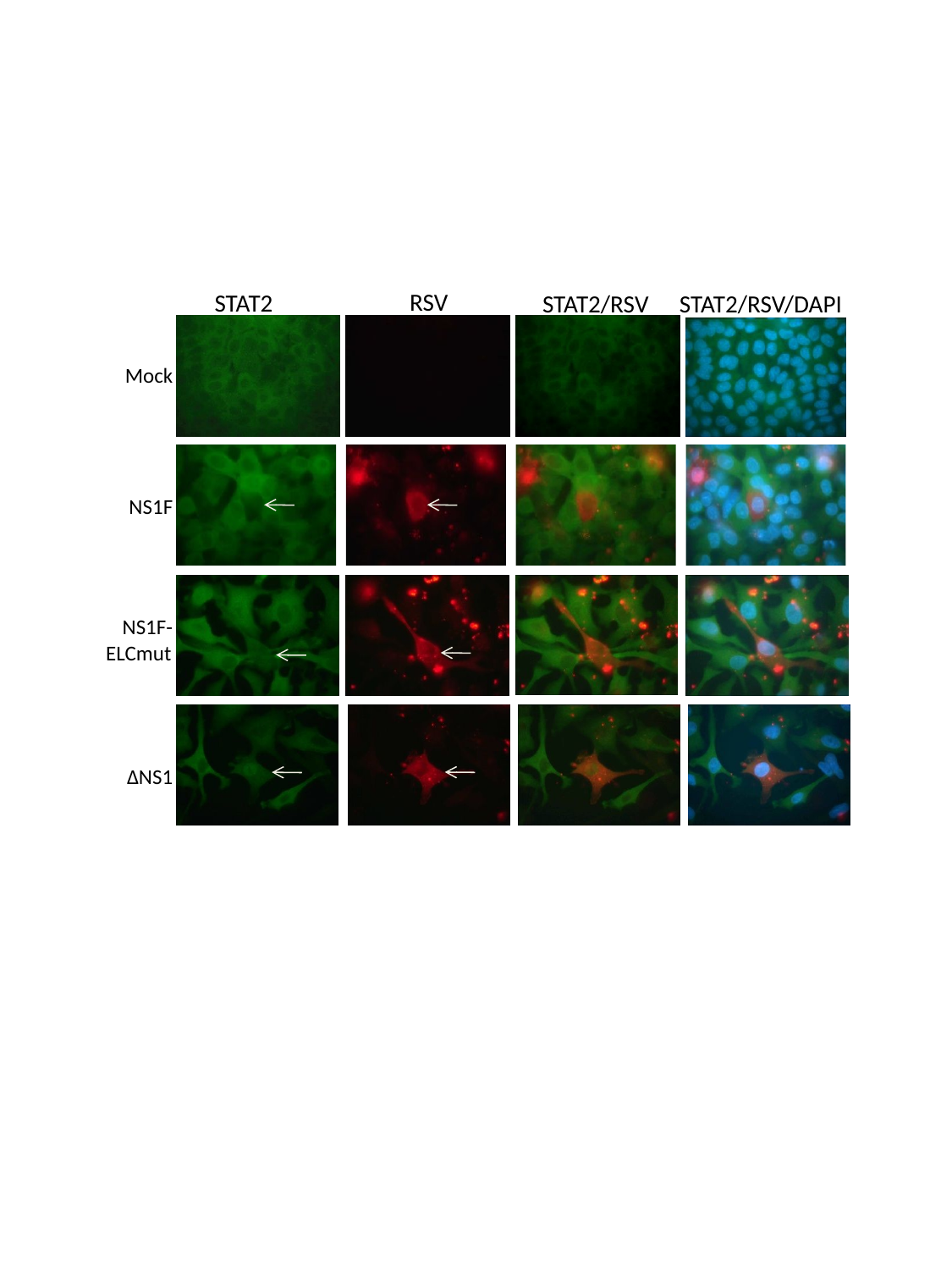

RSV
STAT2
STAT2/RSV/DAPI
STAT2/RSV
Mock
NS1F
 NS1F-
ELCmut
ΔNS1

Supplement: Additional file 1 — Immunofluorescence detection of STAT2 in infected A549 cells. A549 cells were infected with NS1F, NS1F-ELCmut, ΔNS1 or mock infected and fixed 24 h post-infection. STAT2 (green) and RSV (red) were detected using specific antibodies. Nuclei were stained with DAPI and overlay images generated using Photoshop. STAT2 was degraded in NS1F-infected cells. STAT2 was not degraded in NS1F-ELCmut or ΔNS1-infected cells. Arrows = infected cells. [file 1743-422X-8-252-S1.PPT]
